# Reservoir of Bacterial Exotoxin Genes in the Environment

**DOI:** 10.1155/2010/754368

**Published:** 2011-01-09

**Authors:** Veronica Casas, Joseph Magbanua, Gerico Sobrepeña, Scott T. Kelley, Stanley R. Maloy

**Affiliations:** Department of Biology, Center for Microbial Sciences, San Diego State University, 5500 Campanile Drive, San Diego, CA 92182-1010, USA

## Abstract

Many bacteria produce secreted virulence factors called exotoxins. Exotoxins are often encoded by mobile genetic elements, including bacteriophage (phage). Phage can transfer genetic information to the bacteria they infect. When a phage transfers virulence genes to an avirulent bacterium, the bacterium can acquire the ability to cause disease. It is important to understand the role played by the phage that carry these genes in the evolution of pathogens. This is the first report of an environmental reservoir of a bacterial exotoxin gene in an atypical host. Screening bacterial isolates from the environment via PCR identified an isolate with a DNA sequence >95% identical to the *Staphylococcus aureus* enterotoxin A gene (*sea*). 16S DNA sequence comparisons and growth studies identified the environmental isolate as a psychrophilic *Pseudomonas* spp. The results indicate that the *sea* gene is present in an alternative bacterial host, providing the first evidence for an environmental pool of exotoxin genes in bacteria.

## 1. Introduction

Exotoxins are secreted polypeptides produced by certain bacterial pathogens. Many exotoxin genes are carried on mobile genetic elements, including bacterial viruses (bacteriophage or phage). These virulence genes are responsible for many of the symptoms associated with the human disease [1–3]. As highly mobile genetic elements, phage can readily move between different environments, and are generally more resistant to environmental stress than their bacterial counterparts [4–11]. As a result, phage may survive in the environment in reservoirs not yet characterized. Living in these environmental reservoirs, the phage can influence evolution of the bacteria within these environments in several different manners. Horizontal gene transfer between the phage and the bacterium can result in the rapid evolution of new pathogens and may have serious implications in public health [12].

When a phage infects a bacterium, two alternative possibilities may result. One possibility is that the phage can replicate itself using phage and host factors, resulting in lysis of the bacterial host and release of new phage (the lytic lifestyle). Alternatively, the phage can integrate into the bacterial genome and the bacterial host can utilize certain genes the phage carries in its genome for its own benefit (the lysogenic lifestyle) [13]. If a phage encodes virulence genes, such as exotoxin genes, the phage could facilitate the transfer of these genes to nontoxigenic bacterial hosts, thereby increasing the exotoxin gene pool.

The toxins of *Vibrio cholera *(cholera toxin), *Escherichia coli* (shiga toxin), *Corynebacterium diphtheria* (diphtheria toxin), and *Staphylococcus aureus* (enterotoxin A) are encoded by phage [14–17]. The *Staphylococcus *enterotoxin A (*sea*) gene carried by virulent strains of *S. aureus* is encoded by multiple phages, including *ϕ*11, *ϕ*12, *ϕ*13, 80*α*, and 42D [18–21]. Other toxins are carried by phages isolated from *S. aureus *strains from animals, food, and the environment [20, 22–31]. Given that multiple *S. aureus *toxins are encoded by phage, it is possible that multiple transduction events over time have resulted in the generation of the current virulent *S. aureus* strains. An environmental reservoir of toxin genes would provide novel virulence genes, and the genetic exchange between phage and novel bacterial hosts, could provide the mechanism for evolution of novel human pathogens.

To explore this hypothesis, we cultured environmental bacteria and screened them for a phage-encoded exotoxin gene. In this study, we describe the isolation of bacteria from environmental ambient air and the screening of the isolates for the phage-encoded *sea* gene using an exotoxin-specific colony PCR assay. One isolate was confirmed positive for the *sea* gene, and the sequence of the *sea* gene was determined. Using 16S rDNA PCR sequencing and comparison to the nonredundant GenBank nucleotide database, we determined that the environmental isolate was a novel host for the *sea* exotoxin. This is the first report of an alternative bacterial host from the environment that carries a phage-encoded exotoxin gene that is commonly associated with a different bacterial host.

## 2. Results

### 2.1. Cultivation and Exotoxin-Specific (*sea*) PCR Screening of Environmental Isolates

Bacterial isolates were cultivated from the ambient environment by exposing Luria Bertani (LB) plates to air and then incubated at room temperature for 48–72 hr. Eighty nine isolates were subcultured into sterile 96-well plates containing LB with 15% glycerol, and grown with aeration for 48–72 hr. Using colony PCR specific for the *Staphylococcus* enterotoxin A (*sea*) gene, each of the cultivated environmental isolates was screened for the presence of the *sea* gene. Of 89 isolates screened, one putative *sea* positive isolate was identified. The isolate was single colony purified, and the *sea* PCR was repeated on this purified isolate to confirm that the isolate (SEAB3C070426 lab-designated identification) was positive for the *sea* gene. The resulting *sea*-specific PCR product was gel-purified and sequenced.

### 2.2. Characterization of Environmental Isolate

The cultivated and purified environmental isolate “SEAB3C070426” was microbiologically characterized by Gram staining and microscopic evaluation. The environmental isolate was identified as a Gram negative rod. Furthermore, the purified isolate's growth characteristics were evaluated against a *S. aureus *known to carry the *sea* exotoxin gene (*S. aureus *Food Research Institute 913 strain). The environmental isolate did not grow on *S. aureus* enrichment media at 35°C [32], but grew on LB at room temperature after 48–72 hr. In contrast, the *S. aureus* FRI913 control grew on both the enrichment media and LB at both temperatures.

**Figure 1:**
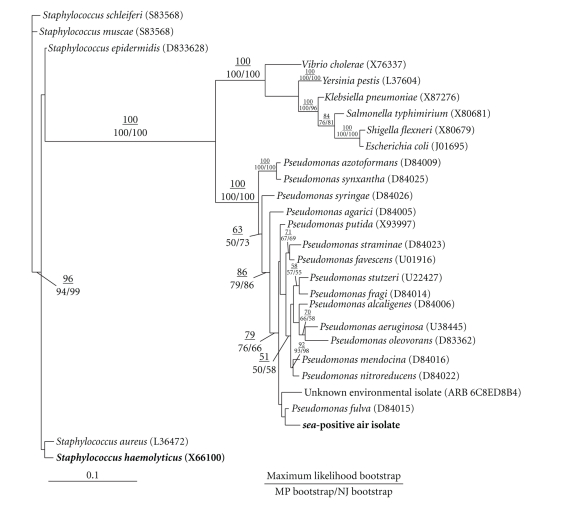
Phylogenetic tree of *sea*-positive isolate, its nearest relatives, and select outgroups. PAUP* was used to generate a consensus phylogenetic tree. Maximum Likelihood (ML) analysis was performed using 100 replicates with 10 random addition sequences searches per replicate. Maximum Parsimony (MP) analysis was performed using searches on 100 bootstrap replicated datasets with 10 search replicates. The Neighbor-joining (NJ) bootstrap analysis was performed with 1000 replicates. The (ML bootstrap)/(MP bootstrap/NJ bootstrap) values are as indicated. The *sea*-positive isolate is highlighted in bold and GenBank Accession numbers of each organism are in parentheses.

To molecularly identify the cultivated environmental isolate carrying the *sea* sequence, 16S rDNA colony PCR was performed and the resulting PCR product was sequenced. The resulting 16S rDNA sequence was imported into the ARB bacterial 16S rDNA database to identify its nearest relatives for downstream phylogenetic analyses [33]. The 16S rDNA PCR product sequence grouped with *Pseudomonas* spp. using the ARB alignment. The nearest relatives identified by ARB were exported and used to generate a phylogenetic tree. The phylogenetic analyses of the ambient air isolate, its nearest relatives, and select outgroups (including *S. aureus*) were performed using the PAUP* program (Figure 1) [33]. The consensus tree generated from Maximum Likelihood (ML), Maximum Parsimony (MP), and Neighbor Joining (NJ) analyses grouped the ambient air isolate with *Pseudomonas* spp., not with *S. aureus*. The ML, MP, and NJ bootsrap values for the main branches of the consensus tree separating the ambient air isolate from *S. aureus* and grouping it with *Pseudomonas* spp. were ≥94. The GenBank Accession number of environmental isolate “SEAB3C070426” is FJ979636.

### 2.3. Characterization of the *sea* PCR Product

**Figure 2:**
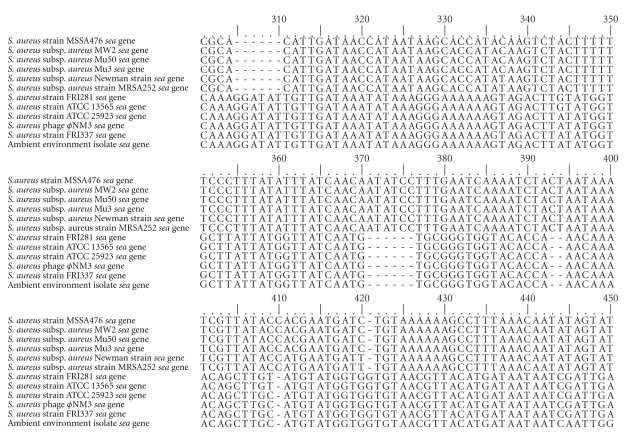
ClustalX2 alignment of top BLASTN hits of the *sea *gene from the ambient environmental isolate. The *sea* PCR product amplified from the cultured ambient air isolate was verified against the GenBank nonredundant database. The FASTA files of the top hits were downloaded and aligned using ClustalX2. The Accession number for the *sea*-related gene is HQ698309.

**Figure 3:**
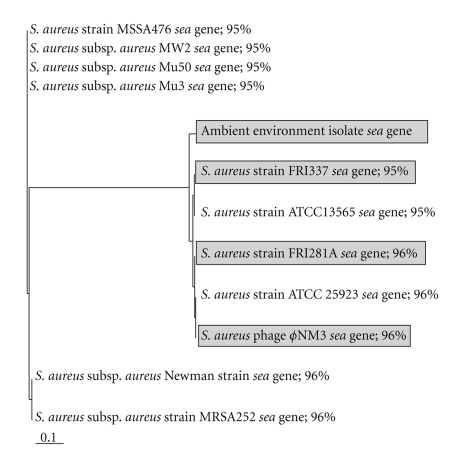
ClustalX2 guide tree of the top BLASTN hits of the *sea* gene from the ambient environmental isolate. The ambient air isolate is highlighted by a gray box along with *S. aureus* strains FRI337 and FRI281A (Food Research Institute) because the positive control used for *sea* exotoxin PCR comes from *S. aureus *strain FRI913. Percent identities are indicated beside the organism name as listed in GenBank. The GenBank Accession number for the *sea*-related gene is HQ698309.

Sequencing of the *sea* PCR product obtained from the environmental isolate generated a 280 bp DNA sequence. The nucleotide composition of the amplified sequence was analyzed using BioEdit [34]. The sequence of the amplified PCR product contained a G + C content of 30% over 92 predicted amino acids. A ClustalW [35] alignment of the translated partial sequence of the *sea*-related gene with known *sea* genes is presented in Figure S1 in Supplemental Material available online at doi: doi:10.1155/2010/754368. A BLASTN alignment of the sequence against the nonredundant GenBank database confirmed that the amplified PCR product shared 95–96% nucleotide sequence identity with known *sea* genes. Most notably, the amplified PCR product was 96% similar to a known *S. aureus* phage, *ϕ*NM3. A multiple alignment of the partial sequence of the *sea*-related gene and guide tree of the top BLASTN hits were produced using ClustalX2 [36] (Figures 2 and 3). BLASTN alignment of the *sea*-related gene against annotated *S. aureus* genomes from The SEED database was also performed [37]. This alignment indicated the *sea*-related gene was related to other *S. aureus *phage-associated enterotoxin genes (supplementary Figure S2). The *sea*-related gene was uploaded to the GenBank database and the Accession number is HQ698309.

## 3. Discussion

Transfer of exotoxin genes to new, as yet uncharacterized, bacterial hosts may facilitate the evolution of novel human pathogens. Many of the exotoxins produced by phage-encoded genes target key eukaryotic cellular processes such as protein synthesis [38–43]. These phage encode the genes responsible for many of the symptoms associated with the human disease. Until recently, most epidemiological and ecological studies of infectious diseases have focused on the presence and activity of the bacteria per se, neglecting the potentially significant role of the phage that carry the exotoxin genes and their role in transmitting these virulence traits [44–47].

Sequence analysis of several environmental metagenomes has shown that phage carrying exotoxin genes are common in the environment, however 16S rDNA analysis of metagenomes from the same environmental samples did not identify the cognate bacterial phage host [48]. A potential explanation of this finding is that phage in the environment may propagate in alternate bacterial hosts rather than those commonly associated with the human disease. The classic textbook description of phage-bacteria interactions implies that phage infection is limited to a specific host. However, as the ecology and physiology of phages has been further investigated, it is clear that some phage can infect multiple bacterial hosts [23, 49–73]. Examples of phage that carry exotoxin genes and can infect alternative hosts include *ctxϕ* (infects both *Vibrio cholerae* and *Vibrio mimicus* [74]), *stx*-2 phage (infects *E. coli* and *Enterobacter cloacae* [75]), and Botulinum toxin E phage (infects *Clostridium botulinum* and *Clostridium butyricum* [76]).

The mechanisms phage have developed to allow infection of a range of hosts are varied. Phage T2 and phage Mu alter their tail fibers to allow infection of alternative hosts [77, 78]. *Bordetella* phage carry diversity generating retroelements that allow the phage to infect bacteria with different cell surface receptors and physiology [79]. Some phage can inactivate or recombine with endogenous lysogens to alter their host range [45, 80, 81]. Inter- and intraspecies examples of phage showing a larger infection range than previously believed have been demonstrated through experimentation with lab strains [52, 62, 65, 67, 71, 72, 82–87].

Infection of alternative hosts in nature has also been demonstrated. Phage isolated from natural marine environments have been shown to subtly influence the composition of the bacterial community of those same environments [88]. Conversely, phage populations from various natural environments such as soil, lake water, and marine sediments have been shown to replicate when incubated with microbes from a different marine environment [6]. Comparison of the distribution of phage types in particular environments with the types of bacteria found in that same environment suggests that phage with quite broad host ranges must exist in nature [89]. Additionally, analyses of uncultured environmental phage libraries have revealed an abundance of mobile elements and genes involved in the mobilization of DNA [90–92]. Altogether these results suggest that phage are capable of infecting different hosts in the environment, providing a major mechanism for the spread of genes between bacteria. Thus, phage may promote promiscuous horizontal gene transfer in nature.

This report provides the first direct evidence that alternative microbial hosts can carry exotoxin genes. The environmental isolate cultivated from outdoor ambient air was confirmed to carry the *sea* exotoxin gene by repeated exotoxin-specific PCR, and by sequencing and alignment of the *sea*-specific PCR product. The *sea*-specific PCR product was 95-96% identical at the nucleotide level to known *sea* genes in the GenBank nonredundant database.

When characterized microbiologically, the cultivated environmental isolate did not share the same characteristics as the control *S. aureus* FRI913 strain known to carry the *sea *gene. It did not grow on *Staphylococcus *enrichment media, nor did it share the same Gram staining properties as the *S. aureus* FRI913 strain (the environmental isolate was a Gram negative rod versus *Staphylococcus* which are Gram positive cocci). These results suggested that the *sea* gene was present in an alternative environmental host.

Further support for this conclusion was provided by sequencing of the 16S rDNA from the cultivated environmental isolate. Alignment of the 16S rDNA sequence from this isolate with the ARB database indicated the isolate was a *Pseudomonas *spp. and did not group with *S. aureus.* Moreover, phylogenetic analyses using PAUP* indicated that the isolate was related to *Pseudomonas spp*, not *S. aureus*. The ML, MP, and NJ bootstrap values of the main branches of the consensus tree that group the isolate with *Pseudomonas* spp. were ≥94, indicating high probability that these branches are robust. This provides robust statistical evidence that the isolate is a *Pseudomonas* spp. Further evidence supporting this identification of the ambient air isolate was its relatively high G + C content (52.36%) like many *Pseudomonas* spp., as compared to the low G + C content of *S. aureus* (32.8%).

We were unable to induce a *sea* positive phage by treatment from the environmental isolate via mitomycin C treatment [81]. There are at least two possible explanations why the environmental isolate was carrying the *sea*-related gene, but did not produce phage. First, generalized transduction could have transferred a region of bacterial chromosomal DNA containing the *sea* gene into the *Pseudomonas* spp. without integration of the phage itself [93]. Second, it is possible that the exotoxin was transferred via a phage, but the phage genes were subsequently mutated due to selection against harmful phage genes in the lysogen [94–102].

**Figure 4:**
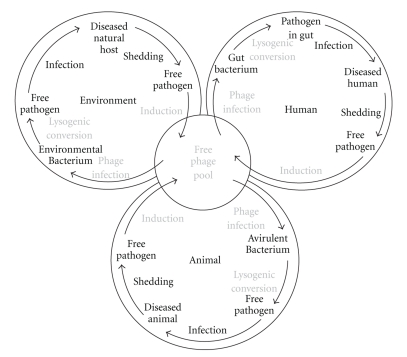
Free phage pool of exotoxin genes. Proposed scenarios for how exotoxin-encoding phage might be maintained in the environment and produce human pathogens through genetic exchange between the free phage pool and the natural, human, and animal environments. Light grey writing indicates phage/host interaction and potential for horizontal gene transfer.

Based on the examples from the literature and the data generated from this study, we propose the following model for transfer of phage-encoded exotoxin genes to a novel bacterium leading to the creation of a new human pathogen. A “free phage pool” (Figure 4) can potentially lead to new disease outbreaks in three ways: (i) transduction of exotoxin genes to an environmental bacterium that subsequently infects a human; (ii) transduction of exotoxin genes from the environmental reservoir to a bacterium in the normal human microbiota; (iii) transduction of a bacterium associated with a nonhuman animal, with subsequent infection of a human. In all three scenarios, once the phage is propagated within the alternate bacterial hosts it can lyse the host and re-enter the “free-phage pool” ready to transduce the exotoxin genes to other bacteria. In this manner, the genes are maintained in the environment independent of the bacterial host typically involved in the human disease.

The evidence that there is an environmental reservoir of exotoxin genes in bacteria that are not normally associated with human disease, suggests the possibility that novel diseases may evolve through horizontal transfer of virulence genes via transduction to new microbial hosts (Figure 4).

## 4. Materials and Methods

### 4.1. Sampling of Indoor and Outdoor Air

To collect bacterial air isolates, Luria Bertani (LB) agar plates containing 50 mg of cyclohexamide to prevent growth of fungi, were exposed to ambient air. These plates were then incubated at room temperature for 48–72 hr. All isolates that grew on the LB plates conditions were then subcultured into sterile 96-well plates containing LB and 15% glycerol. These subcultured isolates were grown, with aeration, at room temperature for 48–72 hr then stored at 4°C until screened by PCR.

### 4.2. PCR Assays and Sequencing

A colony PCR assay was used to initially screen for the Staphylococcus enterotoxin A (*sea*) gene. The *sea*-specific PCR primers amplified a 498 bp partial sequence within the coding region of the *sea* gene. The *sea* primers were (forward primer) 5′-GCAGGGAACAGCTTTAGGC-3′ and (reverse primer) 5′-GTTCTGTAGAAGTATGAAACACG-3′. To identify the bacterial isolate carrying the *sea* gene, a 16S rDNA PCR assay was used and the resulting PCR product sequenced. The primers used for the 16S rDNA PCR were (forward) 5′-AGAGTTTGATCMTGGCTCAG-3′ and (reverse) 5′-TACGGYTACCTTGTTACGACTT-3′. Five microliters of the subcultured isolates suspension was used as a template in the *sea*-specific PCR16S rDNA PCR. The PCR thermocycling conditions for the *sea*-specific PCR and 16S rDNA PCR were as described previously [103]. To control against possible PCR contamination of the environmental sample DNAs, all PCRs were performed in a completely separate room and different building with different air handling systems. The PCR products were run on a 1% agarose gel at 150 V. Sequencing of the *sea*-specific PCR and 16S rDNA PCR products was performed at the SDSU Microchemical Core Facility. To confirm the *sea* PCR product was the target gene, a BLASTN alignment of the PCR product was performed against the nonredundant GenBank database [104]. The top 12 hits were then aligned and a tree generated using ClustalX2 [36].

### 4.3. Microbiological Characterization of Environmental Isolate

The air isolate carrying the *sea*-like gene was characterized by plating onto Staph 110 media [105], a media that enriches for *Staphylococcus aureus*, and LB medium. The plates were incubated at room temperature and at 35°C. A Gram stain was also performed on the isolate identified as *sea*-positive by colony PCR.

### 4.4. Molecular Characterization of Environmental Isolate

Phylogenetic analyses were performed on the 16S rDNA PCR product sequence to molecularly identify the ambient air isolate. An alignment with the ARB 16S rDNA database was used to identify the nearest relatives to the ambient air isolate for use in creating a phylogenetic tree in PAUP* [33]. Maximum Likelihood (ML) was implemented in PAUP* to build the highest likelihood tree under an HKY85 model of sequence evolution with estimated nucleotide frequencies, shape parameter, and number of invariant sites. The heuristic search approach to find the best ML tree included 100 random addition sequence searches using TBR branch swapping. The ML bootstrap involved 100 replicates with 10 random addition sequences searches per replicate. The best Maximum Parsimony (MP) tree was found through a random addition sequence heuristic search strategy with 100 replicates. The maximum number of trees kept during each search was capped at 1000. MP bootstrap analyses were performed using searches on 100 bootstrap replicated datasets using the same heuristic search strategy except with 10, rather than 100, search replicates. The Neighbor-joining (NJ) bootstrap analysis was performed with 1000 replicates.
